# Stereotactic Vertebroplasty for Spinal Metastases with Multilevel Bilateral Pedicle Fractures: A Technical Note

**DOI:** 10.7759/cureus.4123

**Published:** 2019-02-22

**Authors:** Mateo Ziu, Jeffrey I Traylor, Jason Paxman, Boone W Goodgame

**Affiliations:** 1 Neurosurgery, The University of Texas at Austin, Austin, USA; 2 Internal Medicine, The University of Texas at Austin, Austin, USA; 3 Orthopaedics, The University of Texas at Austin, Austin, USA; 4 Oncology, The University of Texas at Austin, Austin, USA

**Keywords:** metastatic spine tumor, vertebroplasty, o-arm, spine, vertebral compression fracture, stereotactic navigation

## Abstract

Vertebral compression fractures (VCFs) represent a significant cause of disability and primarily result from either underlying vertebral body neoplasms or osteoporosis. Vertebroplasty (VP) is a procedure commonly utilized to repair pathologic VCFs in order to manage pain and reinstate vertebral body height. However, there is a paucity of literature on how to manage painful multilevel VCFs with concomitant bilateral pedicle fractures. We describe a patient with a primary prostatic carcinoma and VCFs of the third and fourth lumbar vertebrae (L3 and L4, respectively) with concomitant bilateral pedicle fractures secondary to metastatic disease. Due to the degree of damage to the L3 and L4 vertebral bodies and pedicles, a VP performed via a percutaneous approach was deemed to be too high risk. VP for L3 and L4 was instead performed by utilizing stereotactic spine navigation and an intraoperative O-arm (Medtronic Corporation, Minneapolis, Minnesota). Our result indicates a potential role for stereotactic spine navigation in vertebroplasty for complex pathologic VCFs.

## Introduction

In patients with underlying malignancy, the bones are the third-most common site of primary tumor spread [1]. Of those, approximately two-thirds will have metastases to the spine, putting them at risk for vertebral compression fractures (VCF) that can compromise spinal stability and quality of life [1-2]. Current standard of care for these patients includes the use of kyphoplasty or vertebroplasty (VP) after meeting radiologic and clinical criteria [3-5]. Traditionally, percutaneous VP has been avoided in cases of VCF with concomitant pedicle fracture due to concerns of exacerbating spinal instability. In this technical report, we describe a novel surgical technique using stereotactic spine navigation and an intraoperative O-arm surgical imaging system (Medtronic Corporation, Minneapolis, Minnesota) to perform multilevel VP for a patient with metastatic disease of the third and fourth lumbar vertebrae (L3 and L4, respectively) with concomitant bilateral pedicle fractures. 

## Technical report

**Figure 1:**
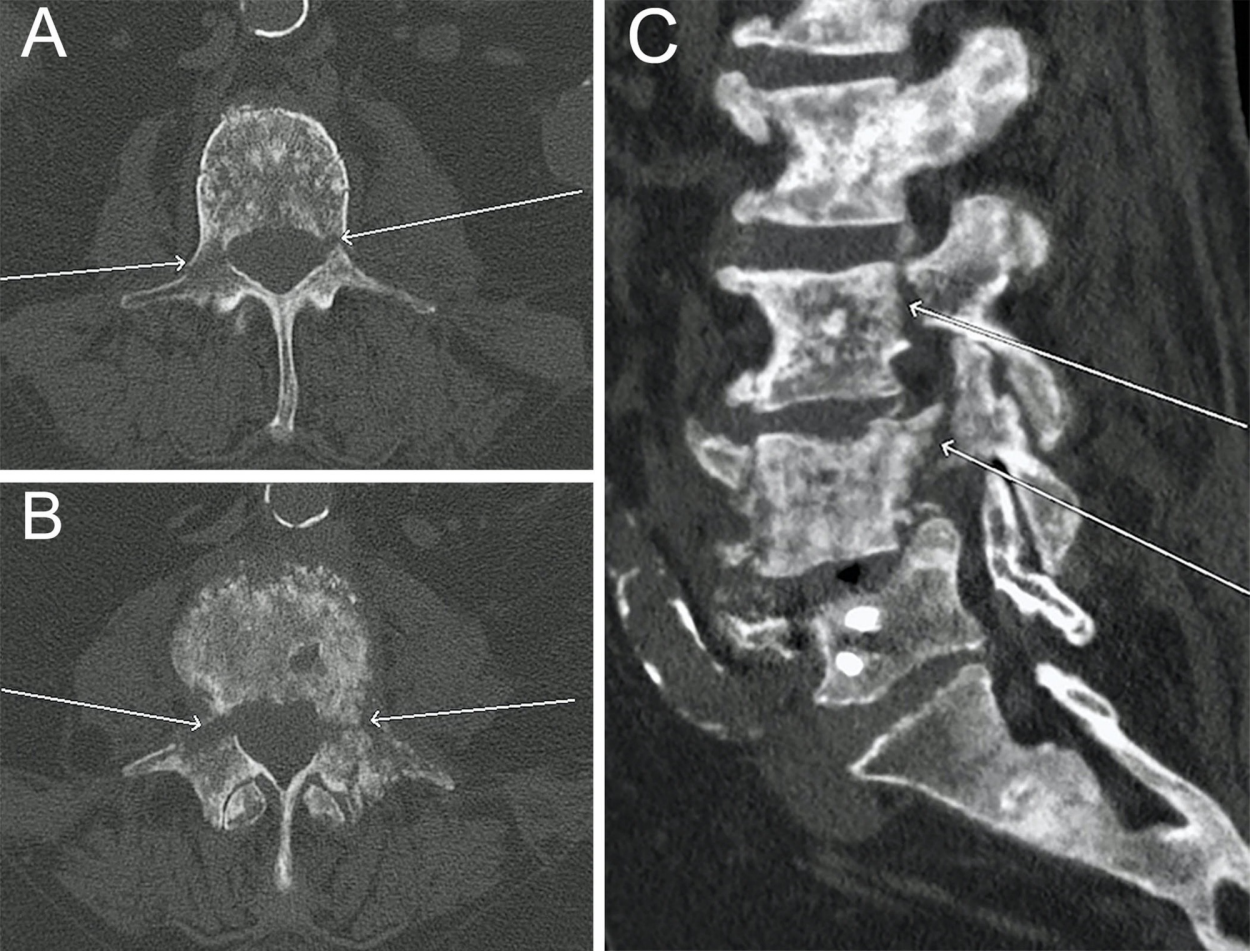
Preoperative computed tomography (CT) images of the lumbar spine (A) and (B): Axial computed tomography (CT) imaging of the lumbar spine demonstrating bilateral pedicle fractures at the third and fourth lumbar levels (L3 and L4, respectively) with pathologic compression fracture at L4. White arrows in both figures point to pedicle fracture lines. (C) Sagittal plane CT revealing pedicle fractures at L3 and L4 (white arrows) and tumor infiltration of the L3 and L4 vertebral bodies.

The patient is a 78-year-old man diagnosed with prostatic carcinoma who presented with severe back pain. Since his diagnosis, he had received surgery, radiation, and chemotherapy for his prostate cancer and bony metastasis, though attempts at controlling his worsening back pain were unsuccessful. Two weeks after percutaneous vertebroplasty (VP) for a metastatic fifth lumbar vertebra (L5) fracture, he arrived in the emergency room (ER) with new debilitating pain. Repeat computed tomography (CT) in the ER showed bilateral L3 and L4 pedicle fractures, tumor infiltration of the corresponding vertebral bodies, and VCF of L4, unseen on previous imaging studies (Figures 1A, 1B, 1C). The patient complained of pain when sitting, standing, and ambulating which was thought to be attributable to metastatic involvement of the vertebral body instead of compromised vertebral stability. All treatment options were discussed by the multidisciplinary oncology team with the patient, including surgical resection of the tumor and instrumented stabilization, VP, medical pain management, and hospice care. After considering several factors, including clinical presentation, images, local and systemic disease burden, patient age, and systemic treatment options, the recommendation was made for palliative VP. Percutaneous VP was thought to increase the risk of instability due to the presence of contiguous, bilateral pedicle fractures. Instead, VP with potential pediculoplasty of the L3 and L4 vertebrae with stereotactic spine navigation and intraoperative O-arm as a palliative treatment for back pain was discussed with the patient and consent was obtained. 

**Figure 2:**
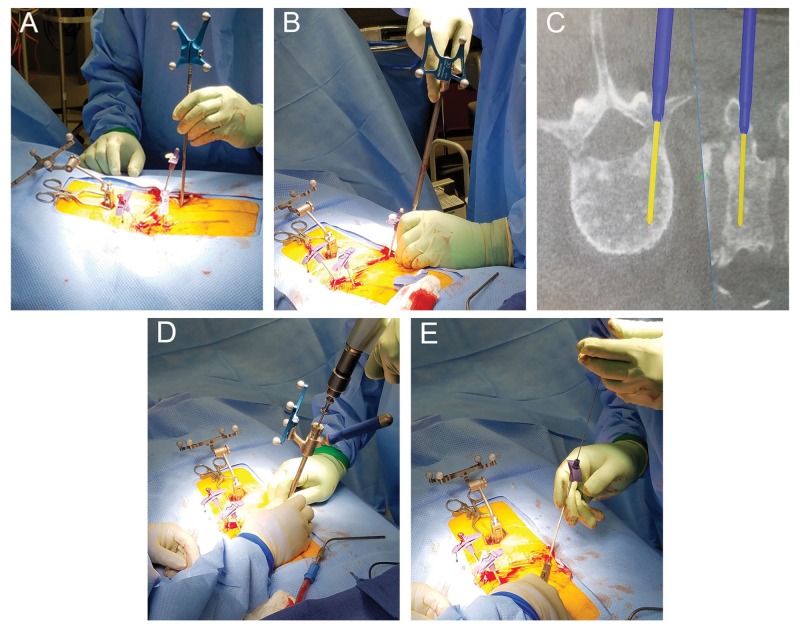
Vertebroplasty (VP) of the fourth lumbar vertebra (L4) using stereotactic spine navigation (A) Utilization of the Stealth probe to determine the entry point into the vertebrae. (B) Introducing a Stealth navigated tubular retractor into the vertebral pedicle. The spine navigation feature of the dilator allows it to be placed in the entry point determined by the Stealth probe. (C) View of the screen on the Stealth system when guiding the Stealth navigated universal drill guide (UDG) through the cannulation established by the dilator. The yellow projection represents the trajectory of the drill, while the light blue outline represents the cannulation left by the dilator retractor. (D) Drilling into the pedicle with the Stealth navigated UDG. The spine navigation feature of the drill guide allows the anticipated trajectory on the Stealth system to be followed throughout the drilling process. (E) Introducing a kyphon cannula over a guidewire placed into the newly drilled hole through the pedicle.

**Figure 3:**
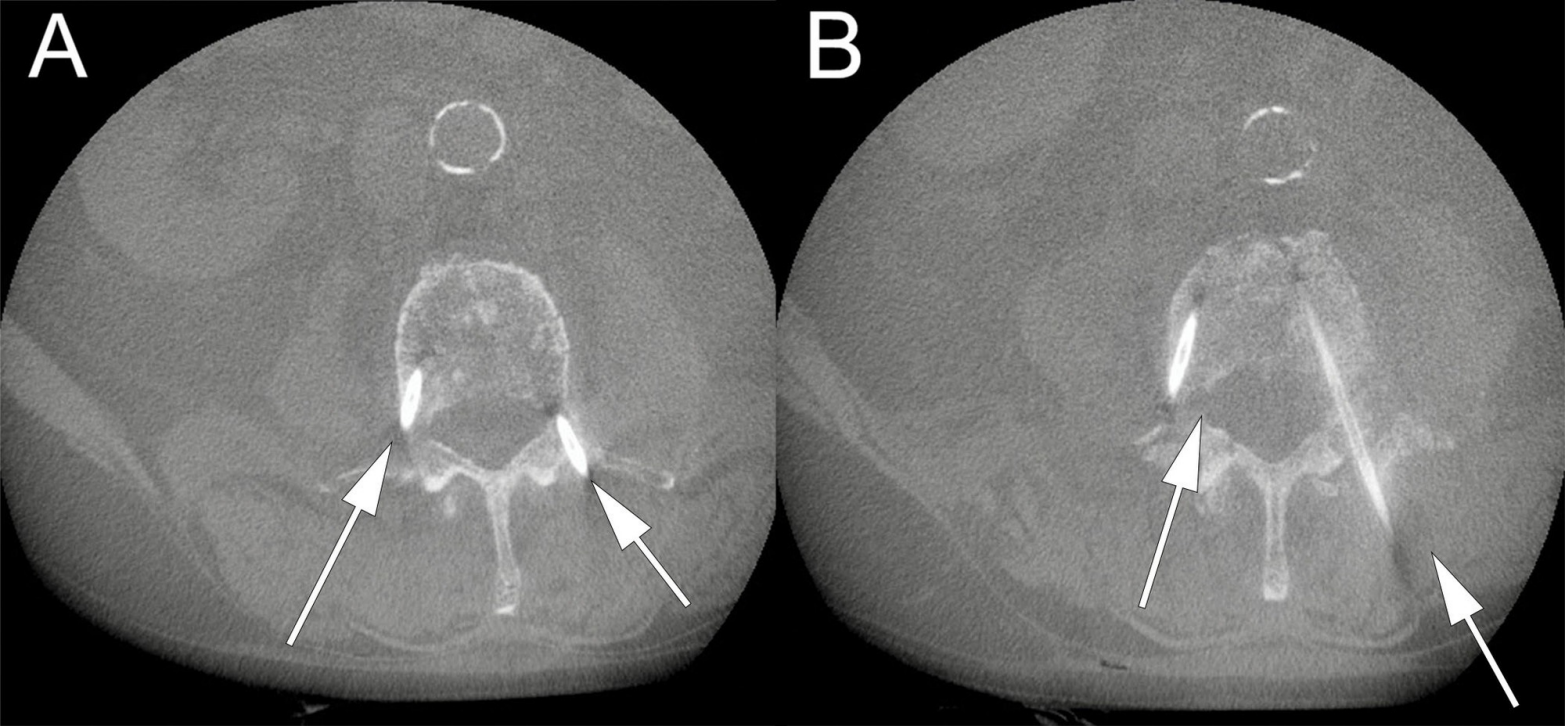
Intraoperative O-arm for kyphon introducer placement confirmation (A) and (B) show the correct positioning of both introducers through the left and right pedicles into the third and fourth vertebral bodies (L3 and L4), respectively. Note that the introducers enter the narrowest point along the pedicle fracture. Arrows point to kyphon cannula introducers.

**Figure 4:**
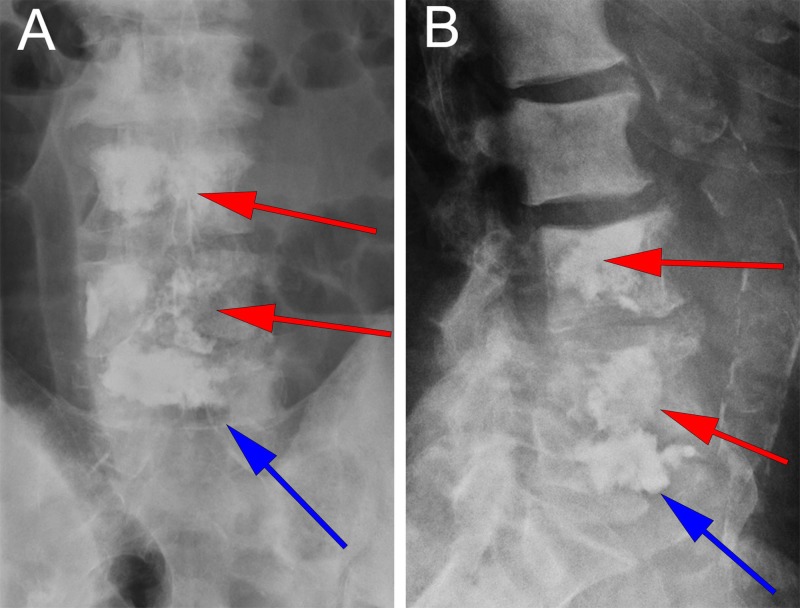
Postoperative lumbar X-Ray Anterior (A) and lateral (B) views demonstrating polymethylmethacrylate (PMMA) cement in the third and fourth lumbar vertebral bodies (L3 and L4, respectively). No extravasation of cement into the spinal canal is noted with normal post-operative changes. Red arrows delineate cement within the L3 and L4 vertebral bodies. Blue arrows delineate extravasated cement from the L4 vertebral body into the L4-L5 disc space.

The patient underwent generalized anesthesia and was positioned supine in the usual fashion. Fluoroscopic X-rays were taken to localize the L3 and L4 vertebrae. The area was prepped and draped in a sterile fashion and a small midline incision was made at the level of the first and second lumbar vertebrae (L1 and L2, respectively) to expose the spinous processes. The Stealth navigation air-frame (Medtronic Corporation, Minneapolis, Minnesota) was clamped to the spinous process of L2 and an O-Arm Surgical Imaging System was used to scan the lumbar spine. The images were then uploaded to the StealthStation Navigation System (Medtronic Corporation, Minneapolis, Minnesota). The trajectories were mapped on the StealthStation software suite with care taken to aim at the narrowest point along the fracture line. Then, using the navigation probe, the entry point into the skin was determined (Figure 2A). Four small incisions were made; two at the entry point to the pedicles of L3, and two at the entry point to the pedicles of L4. Under stereotactic spine navigation, a small Stealth guided tubular dilator retractor (Medtronic Corporation, Minneapolis, Minnesota) was introduced at the location of the left L3 pedicle (Figure 2B). Afterwards a Stealth spine navigation universal drill guide (UDG) (Medtronic Corporation, Minneapolis, Minnesota) was introduced over the dilator. A cordless drill driver with a 3.0 drill bit (Stryker Corporation, Kalamazoo, Michigan) was guided through the Stealth UDG following removal of the tubular retractor. Following the planned trajectory using Stealth guidance (Figure 2C), a pilot hole was created in the pedicle (Figure 2D). A guidewire was introduced through the pilot hole and the drill guide was removed. A Kyphon cannula (Medtronic Corporation, Minneapolis, Minnesota) was then introduced through the guidewire down into the vertebral body (Figure 2E). These steps were repeated on the bilateral pedicles of the L3 and L4 vertebrae. Following placement confirmation of the introducer cannulas through the pedicles of the L3 and L4 vertebrae with the O-Arm (Figures 3A, 3B), the Kyphon cement reservoir was connected to the introducer cannula under fluoroscopic X-ray, and polymethylmethacrylate (PMMA) cement was introduced. During introduction of the cement into the L4 vertebral body, we noticed inferior extravasation of the cement into the disc space of the L4 and L5 vertebrae. Thus, we decided not to proceed with the pediculoplasty to avoid further extravasation into the spinal canal. VP was performed bilaterally at the L3 and L4 vertebrae under fluoroscopic X-ray, with care taken to prevent posterior extravasation. 16 mL of cement was used for the entirety of the L3 and L4 VP. The introducer cannulas were removed, and the skin was closed in usual fashion. There were no acute neurological complications during or immediately after the procedure. Two days later, the patient indicated that his pain had significantly improved, which was attributed to a reduction in oncologic pain. A two-view X-ray of the lumbar spine taken at that time showed normal postoperative changes and no extravasation of the PMMA cement into the spinal canal (Figures 4A, 4B). The patient was discharged from the hospital on postoperative day four in stable condition with improved pain and no neurological deficit to an inpatient rehabilitation facility. One year later, the patient was still alive and ambulating with minimal back pain. Although not completely resolved, the pain remarkably subsided after VP. 

## Discussion

The presence of pedicle fractures makes standard VP procedures more challenging. The thin anatomy of the vertebral pedicles, the variety in terms of degree of fracture separation, as well as poor visualization of the anatomical defect all contribute to difficult procedural repair with standard fluoroscopy guidance. As a result, only a few authors reported treating bilateral pathologic pedicle fractures with percutaneous pediculoplasty [6-7]. However, there are no reports of such repair for bilateral pedicle fracture in multiple, contiguous levels of the lumbar spine. Although this case presented a unique opportunity for a pediculoplasty, the extravasation of the PMMA cement into the inferior disc space in our patient prevented us from proceeding with this approach to avoid leakage into the spinal canal. Even without attempting a pediculoplasty for this patient, the presence and degree of the bilateral pedicle and VCFs in two contiguous vertebrae contributed to a high-risk procedure that prevented utilization of a percutaneous approach VP with a beveled Jamshidi needle, as there was concern that the pressure exerted on the needle could potentially widen the fracture gap.

Singh *et al. *described the utilization of a 10.5 G non-beveled externally threaded needle in a case of bilateral pedicle fractures for pediculoplasty/vertebroplasty, stating that the rotational torque used to advance the needle produces a perpendicular vector force that is easier to control than the classic beveled Jamshidi needles that are pushed by exerting high, difficult-to-control perpendicular force [7]. We believed that by utilizing a high-speed drill, the perpendicular force could be reduced even further, minimizing expansion of the fracture line. Furthermore, the utilization of stereotactic spine navigation allowed us to anticipate the trajectory of the drill prior to entering the pedicle along the narrowest point of the vertebral fracture line, an impossible task using fluoroscopy guidance alone. Though a pediculoplasty was not attempted, the nature of this patient’s bony metastases allowed a unique opportunity for a surgical approach utilizing stereotactic spine navigation as a means of more precise pedicle entrance and cement injection. Thus, we hope this approach to VP has implications on improved pain control in patients with complex, multilevel VCFs.

## Conclusions

With the advent of new stereotactic spine navigational technology and intraoperative CT scanning, VP, via a minimally-invasive surgical approach, may provide a therapeutic alternative for VCFs with concomitant pedicle fractures too complicated for percutaneous repair.
